# Synergistic activity between Triphala and selected antibiotics against drug resistant clinical isolates

**DOI:** 10.1186/s12906-019-2618-1

**Published:** 2019-08-02

**Authors:** Amirthasingam Manoraj, Vasanthi Thevanesam, B. M. R. Bandara, Asela Ekanayake, Veranja Liyanapathirana

**Affiliations:** 1 Department of Ayurveda, Central Province, Kundasale, Sri Lanka; 2 Department of Microbiology, Faculty of Medicine, University of Peradeniya, Peradeniya, Sri Lanka; 3 Department of Chemistry, Faculty of Science, University of Peradeniya, Peradeniya, Sri Lanka

**Keywords:** Triphala, Synergy, Gentamicin, Oxacillin, MDR gram negatives, MRSA

## Abstract

**Background:**

Triphala is an indigenous medical product used for a variety of diseases. This study was conducted to determine the effect of Triphala on antibiotic properties of gentamicin and oxacillin against multi-drug resistant organisms.

**Methods:**

The checkerboard method was used to determine the synergy of Triphala with gentamicin and oxacillin against multi-drug resistant (MDR) Gram negative bacilli and methicillin-resistant *Staphylococcus aureus* (MRSA) using 2,3,5-triphenyltetrazolium chloride (TTC) assay. Fractional inhibitory concentration (FIC) index was calculated.

**Results:**

When tested alone, the minimum inhibitory concentration (MIC) values of gentamicin for Gram negative isolates ranged from 8 to > 64 μg/ml. The MIC values of gentamicin for the Gram negative isolates ranged from 1 to 32 μg/ml when tested with Triphala. The FIC index was < 1 indicating a synergistic interaction in 10 of the 11 isolates and it was 1 indicating an additive effect in one isolate.

The MIC values of oxacillin for MRSA isolates ranged from 4 to > 16 μg/ml with all MICs being equal to or higher than the resistance cut-off level. The MIC level with the addition of Triphala ranged from 0.25 to 4 μg/ml. FIC index was < 1 for all tested isolates indicating a synergistic interaction.

**Conclusions:**

Triphala has synergistic activity with gentamicin against the selected MDR Gram negative bacilli and with oxacillin against MRSA isolates warranting further studies on the possibility of clinical use.

**Electronic supplementary material:**

The online version of this article (10.1186/s12906-019-2618-1) contains supplementary material, which is available to authorized users.

## Background

Triphala is an indigenous medicinal product used in diseases in nose and skin wounds, tumours, cough and upper respiratory diseases, peptic ulcer, mouth ulcers, anemia, abdominal pain, obesity, liver diseases and toxic conditions [1–3]. It is composed of dried pericarp of the fruits of three plants, *Terminalia chebula* Retz. (Combretaceae), *Terminalia bellirica* (Gaertn.) Roxb. (Combretaceae) and *Emblica officinalis* L. (Euphorbiaceae) mixed in equal proportions (1:1:1) [1–4].

The biological activity of Triphala has been studied in some detail, including in animal experiments. Triphala is known to have antimicrobial activity, anti-viral activity, anti-oxidant activity including the ability to scavenge free radicals, anti-inflammatory activity, anti-pyretic activity, wound healing properties, anti-mutagenic and anti-carcinogenic activity as well as hypoglycaemic effects [4–6]. Extracts of Triphala and its individual plants have demonstrated varying degree of activity against a range of Gram negative and Gram positive bacteria [7–10].

Furthermore, Triphala has also shown antibacterial activity against *Enterococcus faecalis* in biofilms formed on dental substrates [11]. A mouthwash prepared from Triphala has been effective in reducing dental plaque formation [12]. Triphala has been incorporated in collagen sponges to reduce wound infection and facilitate healing [13].

Antimicrobial resistance (AMR) is a major issue faced by health care professionals globally. In addition to the identification of novel antibiotics, enhancing the activity of existing antibiotics by combining with novel compounds can also be considered as a strategy to overcome the problem of AMR.

Gentamicin is an aminoglycoside with activity against both Gram negative and positive organisms. Gentamicin is used routinely in combination with other classes of antibiotics in the treatment of different types of bacterial infections. Oxacillin is an indicator drug used to assess sensitivity of *Staphylococcus aureus* to β-lactam antibiotics, the main group of antibiotics used to treat staphylococcal infections unless the isolate is a methicillin-resistant *Staphylococcus aureus* (MRSA). Although synergistic activity of component plants of Triphala with some antibiotics have been studied [14], the synergy between Triphala and gentamicin and oxacillin has not been studied previously to the best of our knowledge. The current study was therefore conducted with the objective of characterizing the antimicrobial activity of Triphala against multi-drug resistant (MDR) organisms and to evaluate synergistic activity with gentamicin and oxacillin against appropriate organisms.

## Methodology

### Preparation of solid extract of Triphala

Specimens of the three constituent plants of Triphala were identified and confirmed by the National Herbarium, Department of National Botanic Gardens, Peradeniya, Sri Lanka and deposited in the National Herbarium with the following tag numbers; *Terminalia chebula* – 522, *Terminalia bellirica* – 816 and *Phyllanthus emblica* – 428 (letter No. 6/01/H/03, dated 2015.11.26 and 2016.01.07).

The dried fruits of *T. chebula*, *T. bellirica* and *P. emblica* bought from a Medicinal shop in Kandy, Sri Lanka were washed with sterile distilled water and crushed to remove the seeds. The pericarp of the three fruits were weighed separately (40 g each), mixed together, to prepare Triphala. An aqueous extract of Triphala was prepared by boiling the Triphala (120 g) in distilled water (400 ml) to 1/8th the initial volume, meeting the conditions of traditional drug preparation. The aqueous extract was freeze-dried and the solid residue was stored in a deep freezer (− 20 °C); the solid residue was used for preparing a dilution series of Triphala as described below.

### Microorganisms

Synergistic activity was tested using 12 MDR pathogens: *Serratia liquefaciens*, *Serratia marcescens*, *Serratia odorifera* biogroup 1, *Proteus* spp., three isolates of *Klebsiella pneumoniae*, *Enterobacter cloacae*, two isolates of *Pseudomonas aeruginosa*, two isolates of *Acinetobacter* spp. and five isolates of Methicillin Resistant *Staphylococcus aureus* (MRSA) obtained from the Department of Microbiology, Faculty of Medicine, University of Peradeniya and Teaching Hospital, Peradeniya. The organisms were stored at − 80 °C (Thermo Scientific freezer) until testing. Antibiotic susceptibility testing was performed for a selected group of drugs using the Clinical and Laboratory Standards Institute [15] recommendations, prior to testing for MIC.

### Preparation of the dilution series of Triphala and antibiotics

The dilution series 5000–4.88 μg/ml of Triphala was prepared using a stock solution of Triphala (10,000 μg/ml) by using double dilution method. Dilution series ranging from 5000 to 9.78 μg/ml was used to test synergistic activity of Triphala and gentamicin against the MDR Gram negatives and a dilution series ranging from 2500 to 4.88 μg/ml was used to test for synergy between Triphala and oxacillin against MRSA.

The dilution series of gentamicin used for synergy testing ranged from 64 to 2 μg/ml. The dilution series of oxacillin used for synergy testing ranged from 32 to 0.5 μg/ml.

### Synergistic activity

Synergistic activity was tested using 2,3,5-triphenyltetrazolium chloride (TTC) assay, using sterile 96 well flat bottomed plates as described previously [16]. Results were read using a colour change.

### Calculation of mean fractional inhibitory concentration (FIC) index

The value of the mean fractional inhibitory concentration (FIC) index as a predictor of synergy was calculated using the recorded MICs [17].

$$ \mathrm{FIC}\ \mathrm{index}={\mathrm{FIC}}_{\left(\mathrm{drug}\ \mathrm{A}\right)}+{\mathrm{FIC}}_{\left(\mathrm{drug}\ \mathrm{B}\right)} $$

$$ \mathrm{FIC}=\mathrm{MIC}\ \mathrm{of}\ \mathrm{drug}\ \mathrm{in}\ \mathrm{combination}/\mathrm{MIC}\ \mathrm{of}\ \mathrm{drug}\ \mathrm{tested}\ \mathrm{in}\mathrm{dividually} $$

The interaction was defined as synergistic if the FIC index was < 1, additive if the FIC index was 1 and antagonistic if the FIC index was > 1 [16, 18].

## Results

All isolates tested were confirmed to be MDR organisms being resistant to three or more classes of antibiotics (Table 1).

**Table 1:** Sensitivity pattern of isolates used in the experiments

Antibiotic	*S. odorifera bio group 1*	*S. marcescens*	*S. liquefaciens*	*E. cloacae*	*K. pneumoniae 1*	*K. pneumoniae 2*	*K. pneumoniae 3*	*Proteus spp.*	*Acinetobacter spp. 1*	*Acinetobacter spp. 2*	*P. aeruginosa 1*	*P. aeruginosa 2*
Cefotaxime/Ceftazidime	R/R	R/R	S/S	R/R	R/R	R/R	R/R	R/R	R/R	R/R	NA/R	NA/R
Imipenem/Meropenem	S/S	R/R	R/S	S/R	R/R	S/S	R/R	R/R	R/R	R/R	S/R	S/R
Gentamicin/Amikacin	R/S	R/S	R/I	R/R	R/R	R/S	R/I	R/R	R/R	R/R	R/R	R/R
Ciprofloxacin	R	R	R	R	R	I	R	R	R	R	R	I
Tetracycline	S	R	R	R	R	R	S	R	R	R	NA	NA
Trimethoprim-sulfamethoxazole	R	R	R	R	R	R	R	R	R	R	NA	NA
Aztreonam	R	R	I	R	R	R	R	R	NA	NA	R	R

*S* Sensitive, *R* Resistant, *I* Intermediate

### MIC values when tested alone and in combination

When tested alone, the MIC values of gentamicin for the Gram negative isolates ranged from 8 to > 64 μg/ml (Table 2 and Additional file 1: Table S1). All enterobacteriaceae isolates had MICs within the resistant range to gentamicin (≥ 16 μg/ml). The two acinetobacter spp. tested also had MICs that were considered resistant (≥ 16 μg/ml). One of the pseudomonads tested had a sensitive MIC value despite demonstrating a resistant zone diameter in the disc diffusion testing. The MIC values of gentamicin for these Gram negative isolates ranged from 1 to 32 μg/L on testing with Triphala. Six of the 11 isolates with MICs demonstrating resistance to gentamicin when tested alone had MICs in the sensitive range when tested with Triphala while four isolates had their MICs lowered to the intermediate sensitive level. One isolate remained resistant despite demonstrating a two-fold drop in the MIC.

**Table 2:** Mean MICs of gentamicin and Triphala for MDR Gram negatives, when tested alone and in combination

Isolate	Mode MIC for gentamicin alone μg/ml	Interpretation	Mode MIC for gentamicin when tested with Triphala μg/ml	Interpretation	Mean concentration of Triphala at which synergy was shown μg/ml
*S. liquefaciens*	> 64	R	1	S	2500
*S. odorifera* biogroup 1	64	R	8	I	625
*S. marcescens*	> 64	R	32	R	625
*Proteus* spp.	> 64	R	1	S	312
*K. pneumonia* 1	64	R	8	I	1250
*K. pneumonia* 2	32	R	1	S	1250
*K. pneumonia* 3	32	R	1	S	2500
*E. cloacae*	64		8	I	2500
*Acinetobacter* spp. 1	> 64	R	2	S	625
*Acinetobacter* spp. 2	> 64	R	8	I	156
*P. aeruginosa* 1	32	R	16	R	1250
*P. aeruginosa* 2	8	S	4	S	78

*S* Sensitive, *R* Resistant, *I* Intermediate

The MIC values of oxacillin for the MRSA isolates ranged from 4 to > 16 μg/ml with all MICs being equal to or higher than the resistance cut-off level. The MIC level with the addition of Triphala ranged from 0.25 to 4 μg/ml. Four out of the five isolates demonstrated a drop in the MIC to the sensitive range (Table 3 and Additional file 1: Table S2).

**Table 3:** Mean MICs of oxacillin and Triphala for MRSA isolates, when tested alone and in combination

Isolate	Mode MIC for oxacillin alone (μg/ml)	Interpretation	Mode MIC for oxacillin when tested with Triphala (μg/ml)	Interpretation	Mean concentration of Triphala at which synergy was shown (μg/ml)
MRSA 1	> 16	R	1	S	78
MRSA 2	4	R	0.25	S	78
MRSA 3	> 16	R	0.25	S	78
MRSA 4	> 16	R	4	R	39
MRSA 5	16	R	2	S	78

*S* Sensitive, *R* Resistant

The FIC indices were calculated for gentamicin–Triphala and oxacillin-Triphala combinations (Table 4). Eleven of the twelve gentamicin-Triphala interactions and all oxacillin-Triphala interactions were synergistic while one gentamicin-Triphala interaction, for a *Pseudomonas aeruginosa* isolate was additive in nature. None of them were antagonistic.

**Table 4:** FIC index of antibiotic (gentamicin/oxacillin) and Triphala for the MDR bacterial isolates

Isolate	FIC of antibiotic	FIC of Triphala	FIC index	Interpretation
Gentamicin and Triphala				
*S. liquefaciens*	< 0.016	< 0.25	< 0.266	Synergistic
*S. odorifera* biogroup 1	0.125	0.25	0.375	Synergistic
*S. marcescens*	< 0.5	0.25	< 0.75	Synergistic
*Proteus* spp.	< 0.016	0.25	< 0.27	Synergistic
*K. pneumonia* 1	0.125	0.25	0.375	Synergistic
*K. pneumonia* 2	0.031	0.25	0.281	Synergistic
*K. pneumonia* 3	0.031	0.5	0.531	Synergistic
*E. cloacae*	0.125	0.5	0.625	Synergistic
*Acinetobacter* spp. 1	< 0.031	0.25	< 0.281	Synergistic
*Acinetobacter* spp. 2	< 0.125	0.061	< 0.186	Synergistic
*P. aeruginosa* 1	0.5	0.5	1	Additive
*P. aeruginosa* 2	0.5	0.13	0.63	Synergistic
Oxacillin and Triphala				
MRSA 1	< 0.06	0.25	< 0.31	Synergistic
MRSA 2	0.06	0.5	0.56	Synergistic
MRSA 3	< 0.016	0.5	< 0.516	Synergistic
MRSA 4	< 0.25	0.25	< 0.5	Synergistic
MRSA 5	0.125	0.5	0.625	Synergistic

## Discussion

In the current study, we demonstrated that Triphala interacts in a synergistic manner with gentamicin against the selected MDR Gram negative bacilli and with oxacillin against MRSA. Aminoglycosides including gentamicin act by interfering with bacterial protein synthesis. Gentamicin has been used widely in combination with other β-lactam antibiotics [19]. Non-lethal damage by theβ-lactam antibiotics to the cell wall of bacteria is considered to facilitate the entry of the aminoglycoside antibiotic to the bacterial cell, enhancing its killing ability [20]. Aminoglycoside resistance, including resistance to gentamicin occurs through enzymatic deactivation of the drug, active efflux of the drug or cell wall changes leading to reduced uptake of the drug and decreased binding of the drug to the 16S ribosome due to mutations or methylation [19]. Both ethanolic and aqueous extracts of Triphala contain phenolics, flavonoids and carotenoids [21]. Antibacterial activity of Triphala has been shown to correlate to its phenolic content [22]. Phenolic compounds are known to disrupt the cell membranes of bacteria [23]. Therefore, the mechanism of synergy between gentamicin and Triphala may be similar to that between gentamicin andβ-lactams.

Oxacillin acts by interfering with cell wall synthesis. The main mechanism of resistance to all β-lactams in MRSA is the presence of PBP2a that has a lower affinity to this group of drugs [24]. Synergy between phenolic compounds and oxacillin against MRSA, similar to our findings, has been documented previously [25, 26].

The chemical structures of active phenolic compounds determine their antibacterial activity [23]. Therefore, it is important that the active phytochemicals of Triphala be identified and their structures elucidated in furthering this study.

While synergism or additive activity was noted across all antimicrobial-plant compounds tested in the study, not all gentamicin MIC drops resulted in achieving clinical sensitive breakpoints. Furthermore, no consistency has been observed in the reversion of gentamicin MIC to sensitive levels across the organisms tested. Presence of varying mechanisms of resistance among the test isolates may have contributed to this. As the mechanisms of resistance for the test isolates were not established, we are unable to further validate this claim.

While there was a drop in the MIC of Triphala against all organisms tested when used in combination with the antibiotics, the values remained relatively high (original MIC values not shown). However, the reported MIC values for Triphala and its constitutive plant products when tested alone has ranges similar to that obtained in our study [22, 23]. Similar levels have been reported for other plant products as well [27, 28].

Aminoglycoside use had increased in the recent past for the treatment of infections caused by MDR organisms as the rate of emergence of resistance to aminoglycosides has been low. However, the reason for discontinuation of the aminoglycoside in many instances is nephrotoxicity [29]. Interestingly, Triphala has renal protective effects both in animal studies [30] and limited clinical studies [31], presumably due to its antioxidant properties. In vitro and in-vivo studies of Triphala and gentamicin together to explore the possible benefits are warranted in future.

## Conclusion

In conclusion, Triphala demonstrated synergistic activity with gentamicin against selected MDR Gram negative bacilli and with oxacillin against MRSA, warranting further studies to identify the possibility of using such combinations in clinical practice.

## Additional file

Additional file 1: **Table S1**. Result of the triplicates - the MICs of gentamicin and Triphala for MDR Gram negatives, when tested alone and in combination. **Table S2**. Result of the triplicates -MICs of oxacillin and Triphala for MRSA isolates, when tested alone and in combination. (DOC 60 kb)

## Data Availability

The datasets used and/or analysed during the current study are available from the corresponding author on reasonable request.

## Abbreviations

AMR: Antimicrobial resistance
FIC: Fractional inhibitory concentration
MDR: Multi drug resistant
MIC: Minimum inhibitory concentration
MRSA: Methicillin Resistant *Staphylococcus aureus*
TTC: 2,3,5-Triphenyltetrazolium chloride
