# Supplementary material for: Synergistic activity between Triphala and selected antibiotics against drug resistant clinical isolates
Source: BMC Complement Altern Med. 2019 Aug 2;19:199. doi: 10.1186/s12906-019-2618-1 (PMC6679468; doi:10.1186/s12906-019-2618-1)
Supplement: Supplementary file 1 — Table S1. Result of the triplicates - the MICs of gentamicin and Triphala for MDR Gram negatives, when tested alone and in combination. Table S2. Result of the triplicates -MICs of oxacillin and Triphala for MRSA isolates, when tested alone and in combination. (DOC 60 kb) [file 12906_2019_2618_MOESM1_ESM.doc]

| Supplemenrary Table 1. Result of the triplicates - the MICs of gentamicin and Triphala for MDR Gram negatives, when tested alone and in combination | | | | | | | | | | | | |
| --- | --- | --- | --- | --- | --- | --- | --- | --- | --- | --- | --- | --- |
| Isolate | Mean MIC for Triphala alone  µg/ml | | | Mean MIC for gentamicin alone µg/ml | | | Mean MIC for gentamicin when tested with Triphala µg/ml | | | Mean concentration of Triphala at which synergy was shown µg/ml | | |
|  | **1** | **2** | **3** | **1** | **2** | **3** | **1** | **2** | **3** | **1** | **2** | **3** |
| *S. liquefaciens* | >5000 | >5000 | >5000 | > 64 | > 64 | > 64 | 1 | 1 | 1 | 2500 | 2500 | 2500 |
| *S. odorifera* biogroup 1 | 2500 | 2500 | 2500 | 64 | 64 | > 64 | 8 | 8 | 8 | 625 | 625 | 625 |
| *S. marcescens* | 1250 | 1250 | 1250 | > 64 | > 64 | > 64 | 32 | 32 | 32 | 625 | 625 | 625 |
| *Proteus* spp. | 625 | 625 | 625 | > 64 | 64 | > 64 | 1 | 1 | 1 | 312 | 312 | 312 |
| *K. pneumonia* 1 | 2500 | 2500 | 2500 | >64 | 64 | 64 | 8 | 4 | 8 | 1250 | 2500 | 1250 |
| *K. pneumonia* 2 | 2500 | 2500 | 2500 | 32 | 32 | 32 | 1 | 1 | 1 | 1250 | 1250 | 1250 |
| *K. pneumonia* 3 | 5000 | 5000 | 5000 | 32 | 32 | 16 | 1 | 1 | <1 | 2500 | 2500 | 2500 |
| *E. cloacae* | 5000 | 5000 | 5000 | 64 | 64 | 64 | 4 | 8 | 8 | 2500 | 2500 | 2500 |
| *Acinetobacter* spp. 1 | 1250 | 1250 | 1250 | 64 | > 64 | > 64 | 2 | 2 | 2 | 312 | 625 | 625 |
| *Acinetobacter* spp. 2 | 312 | 312 | 312 | > 64 | > 64 | > 64 | 8 | 8 | 8 | 156 | 156 | 156 |
| *P. aeruginosa* 1 | 2500 | 2500 | 2500 | 32 | 32 | 32 | 16 | 16 | 16 | 1250 | 1250 | 1250 |
| *P. aeruginosa* 2 | 625 | 625 | 625 | 8 | 8 | 8 | 4 | 4 | 4 | 78 | 78 | 78 |

Supplemenrary Table 2. Result of the triplicates -MICs of oxacillin and Triphala for MRSA isolates, when tested alone and in combination.

| Isolate | Mean MIC for Triphala alone  (µg/ml) | | | Mean MIC for oxacillin alone (µg/ml) | | | Mean MIC for oxacillin when tested with Triphala (µg/ml) | | | Mean concentration of Triphala at which synergy was shown (µg/ml) | | |
| --- | --- | --- | --- | --- | --- | --- | --- | --- | --- | --- | --- | --- |
|  | **1** | **2** | **3** | **1** | **2** | **3** | **1** | **2** | **3** | **1** | **2** | **3** |
| MRSA 1 | 156 | 156 | 156 | > 16 | > 16 | > 16 | 1 | 1 | 1 | 78 | 78 | 78 |
| MRSA 2 | 156 | 156 | 156 | 4 | 4 | 4 | 0.25 | 0.25 | 0.25 | 78 | 78 | 78 |
| MRSA 3 | 156 | 156 | 156 | > 16 | > 16 | > 16 | 0.25 | 0.25 | 0.25 | 78 | 78 | 78 |
| MRSA 4 | 156 | 156 | 156 | > 16 | > 16 | > 16 | 4 | 4 | 4 | 39 | 39 | 39 |
| MRSA 5 | 156 | 156 | 156 | 16 | 16 | 16 | 2 | 2 | 2 | 78 | 78 | 78 |
